# Supplementary material for: An Amino Acid Mixture to Counteract Skeletal Muscle Atrophy: Impact on Mitochondrial Bioenergetics
Source: Int J Mol Sci. 2024 May 31;25(11):6056. doi: 10.3390/ijms25116056 (PMC11173258; doi:10.3390/ijms25116056)
Supplement: Supplementary file 1 [file ijms-25-06056-s001.zip › ijms-3006793-supplementary.pdf]

Supplementary Material

An amino acid mixture to counteract skeletal muscle atrophy: impact on mitochondrial bioenergetics

Francesco Bellanti, Aurelio Lo Buglio, Giuseppe Pannone, Maria Carmela Pedicillo, Ilenia Sara De Stefano, Angela Pignataro, Cristiano Capurso and Gianluigi Vendemiale

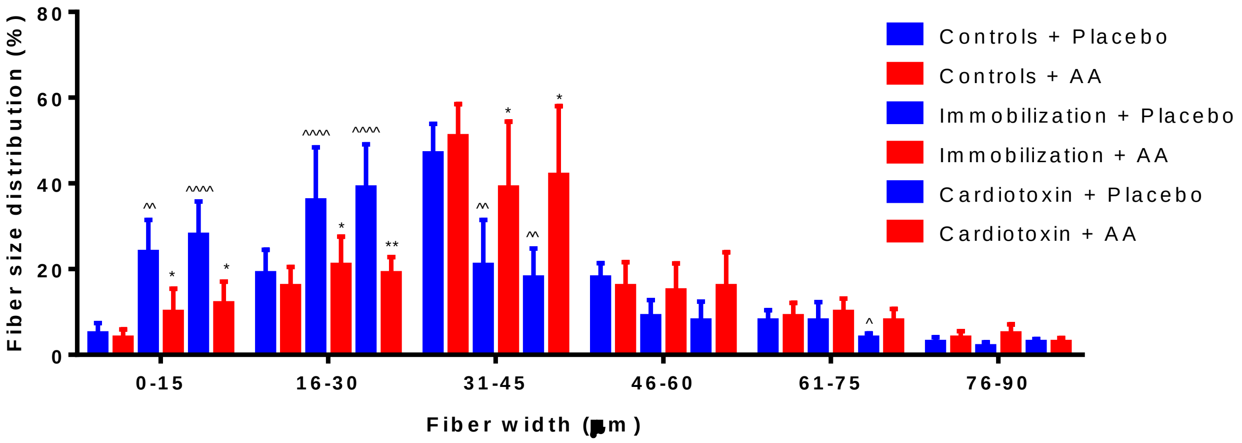

Figure S1. Impact of AA on skeletal muscle fiber size distribution. Data are expressed as mean ± standard deviation of 5 different experiments. Statistical differences were assessed by two-way analysis of variance (ANOVA) and Tukey as post hoc test. ^ = p < 0.05 vs control groups; ^^, p < 0.01 vs control groups; ^^^^ = p < 0.0001 vs control groups. * = p < 0.05 vs placebo groups; ** = p < 0.01 vs placebo groups.

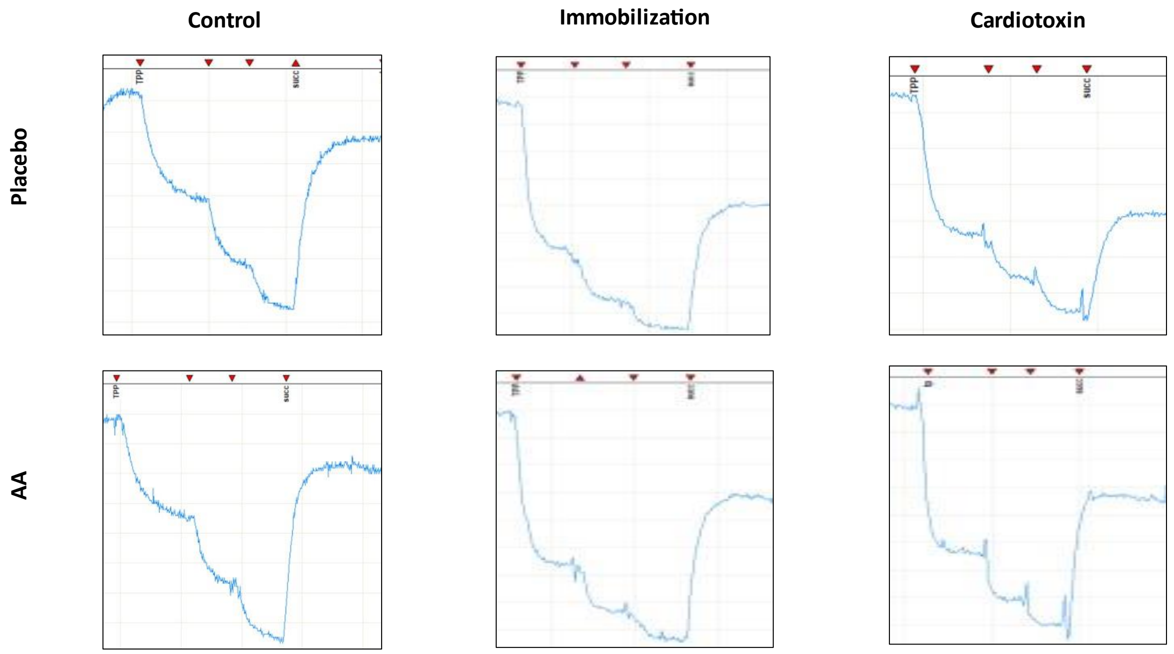

Figure S2. Representative curves of membrane potential (MMP) measurement in freshly isolated skeletal muscle mitochondria from all the study models. MMP was measured by adding 10 mM succinate (succ) after three consecutive additions of 1 μM tetraphenylphosphonium (TPP) to calibrate the TPP electrode. AA, amino acid mixture.

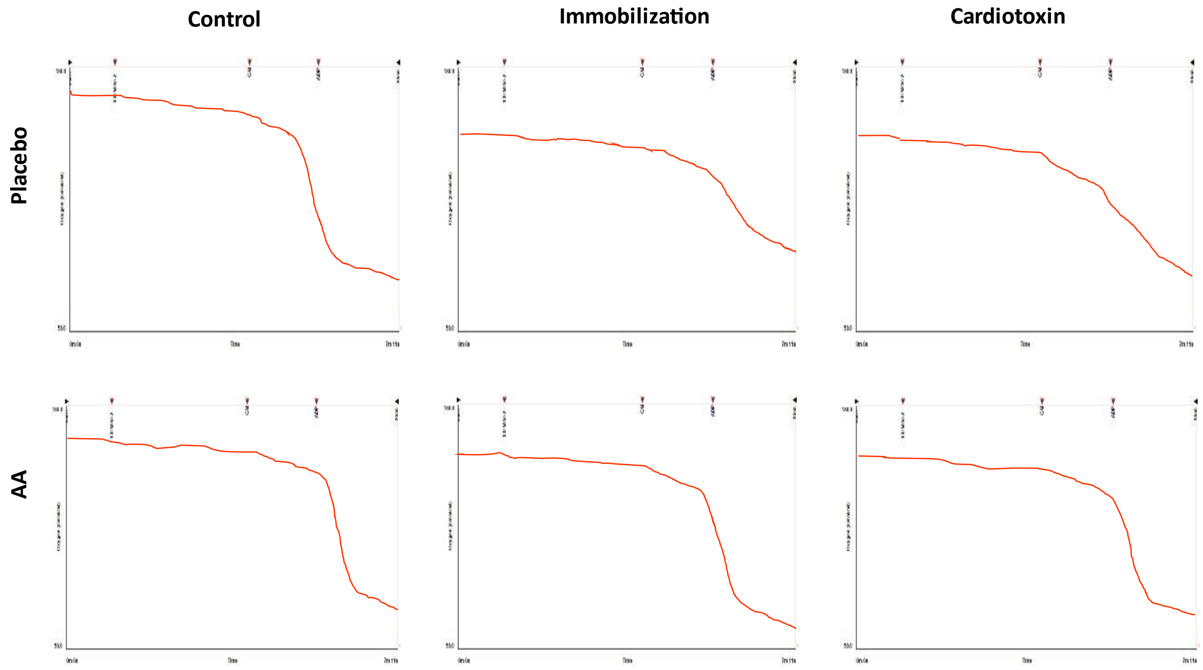

Figure S3. Representative polarographic curves of Complex I-related oxygen consumption rate measurement in freshly isolated skeletal muscle mitochondria from all the study models. GM, glutamate-malate; AA, amino acid mixture.

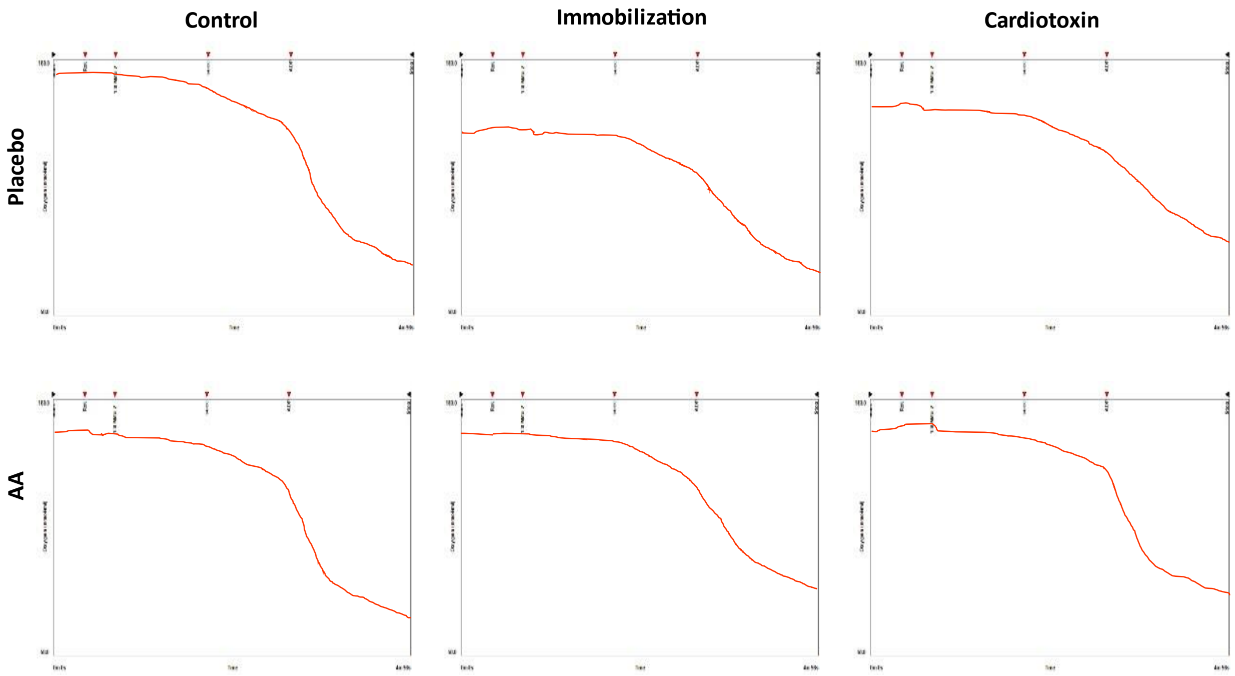

Figure S4. Representative polarographic curves of Complex II-related oxygen consumption rate measurement in freshly isolated skeletal muscle mitochondria from all the study models. Succ, succinate; AA, amino acid mixture.

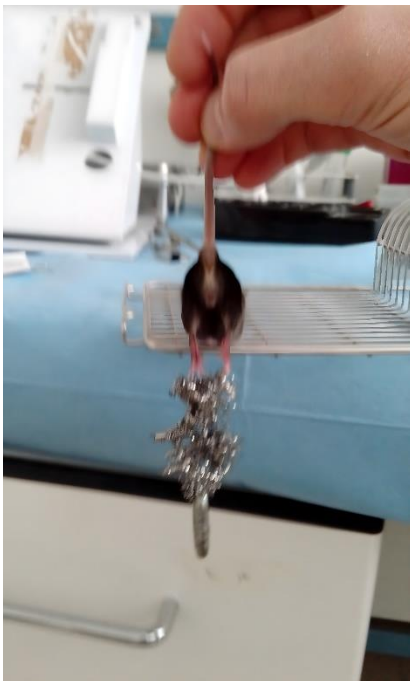

Figure S5. Representative image of hindlimb grip strength test.
